# EZH2 overexpression is associated with poor prognosis in patients with glioma

**DOI:** 10.18632/oncotarget.13478

**Published:** 2016-11-21

**Authors:** Yanyang Zhang, Xinguang Yu, Ling Chen, Zhibin Zhang, Shiyu Feng

**Affiliations:** ^1^ Department of Neurosurgery, Chinese PLA General Hospital, Beijing 100853, China

**Keywords:** EZH2, glioma, biomarker, prognosis

## Abstract

Previous studies have investigated the prognostic value of enhancer of zeste homolog 2 (EZH2) expression in patients with glioma but conclude contradictory results. We aimed to comprehensively evaluate the prognostic role of EZH2 in glioma by meta-analysis. The databases of PubMed, Embase and Web of Science were searched. Hazard ratio (HR) and 95% confidence interval (CI) were combined to assess the association between EZH2 and overall survival (OS) as well as progression-free survival (PFS). Odd ratio (OR) and 95% CI were calculated to investigate the relevance of EZH2 on clinical factors. Six studies with 575 patients were included for meta-analysis. The results showed that EZH2 overexpression was correlated with poor OS (*n* = 6, HR = 2.23, 95% CI: 1.56–3.19, *p* < 0.001) and PFS (*n* = 3, HR = 2.23, 95% CI: 1.56–3.19, *p* < 0.001). Subgroup analysis showed that EZH2 had enhanced prognostic value in Asian patients, for WHO grade I-IV and when using immunohistochemistry (IHC) method. In addition, EZH2 was associated with KPS score < 80. No evidence of publication bias was found in this meta-analysis. In conclusion, the present study showed that EZH2 was a potential prognostic marker for poor OS, PFS and lower KPS score in glioma patients.

## INTRODUCTION

Brain tumors represent 1.9% of all new cancer cases and account for 2.3% of cancer related deaths globally [1]. Glioma, approximately comprising 80% of all primary malignant brain tumors, is the most prevalent type and results in disappointing survival outcomes [2]. Gliomas are classified as four histopathological grades according to World Health Organization (WHO), ranging from grade I to grade IV. In terms of treatment, the treatment approaches mainly include surgical resection, radiotherapy, chemotherapy and multiple therapies in combination for glioma [3]. Although much progresses have been achieved in glioma therapies, the prognosis of glioma is still frustrating, with most grade IV glioblastoma patients only surviving for less than 2 years [4]. Therefore, it is necessary to identify molecular prognostic markers to help to tailor therapeutic regimens and to predict clinical outcomes of high risk patients.

Enhancer of zeste homolog 2 (EZH2) is the core structural component of polycomb repressive complexes 2 (PRC2), which can silence tumor suppressor genes through methylating lysine 27 of histone 3 (H3K27)[5]. Moreover, EZH2 is pivotal to maintain the undifferentiated status of neuroblastoma by epigenetic repression of various tumor suppressor genes including CASZ1, CLU, RUNX3, and NGFR [6]. Previous evidence showed that EZH2 promoted cancer cells to proliferate, metastasis and invade [7]. Furthermore, EZH2 plays a tumorigenic role by epigenetic activation of oncogenic signaling pathways and through promotion of tumor angiogenesis [8]. Growing evidence showed that EZH2 is upregulated in a wide spectrum of solid tumors including non-small cell lung cancer[9], prostate cancer [10], head and neck squamous cell carcinoma [11], breast cancer [12], liver cancer [13], and gastric cancer [14]. A variety of studies also investigate the prognostic role of EZH2 in glioma patients [15–20], whereas the results remains inconsistent. For example, Wu *et al.* [15] identified EZH2 overexpression as a prognostic marker for shorter overall survival (OS) in glioma patients receiving surgical resection. However, Ailon and colleagues [16] failed to find any association between EZH2 and OS in glioma in their study. In order to give a clarification for this issue, we thus collected most recent data and employed a meta-analysis to pool the results from eligible studies to present a quantitative estimation.

## RESULTS

### Study selection and characteristics

**Figure 1:**
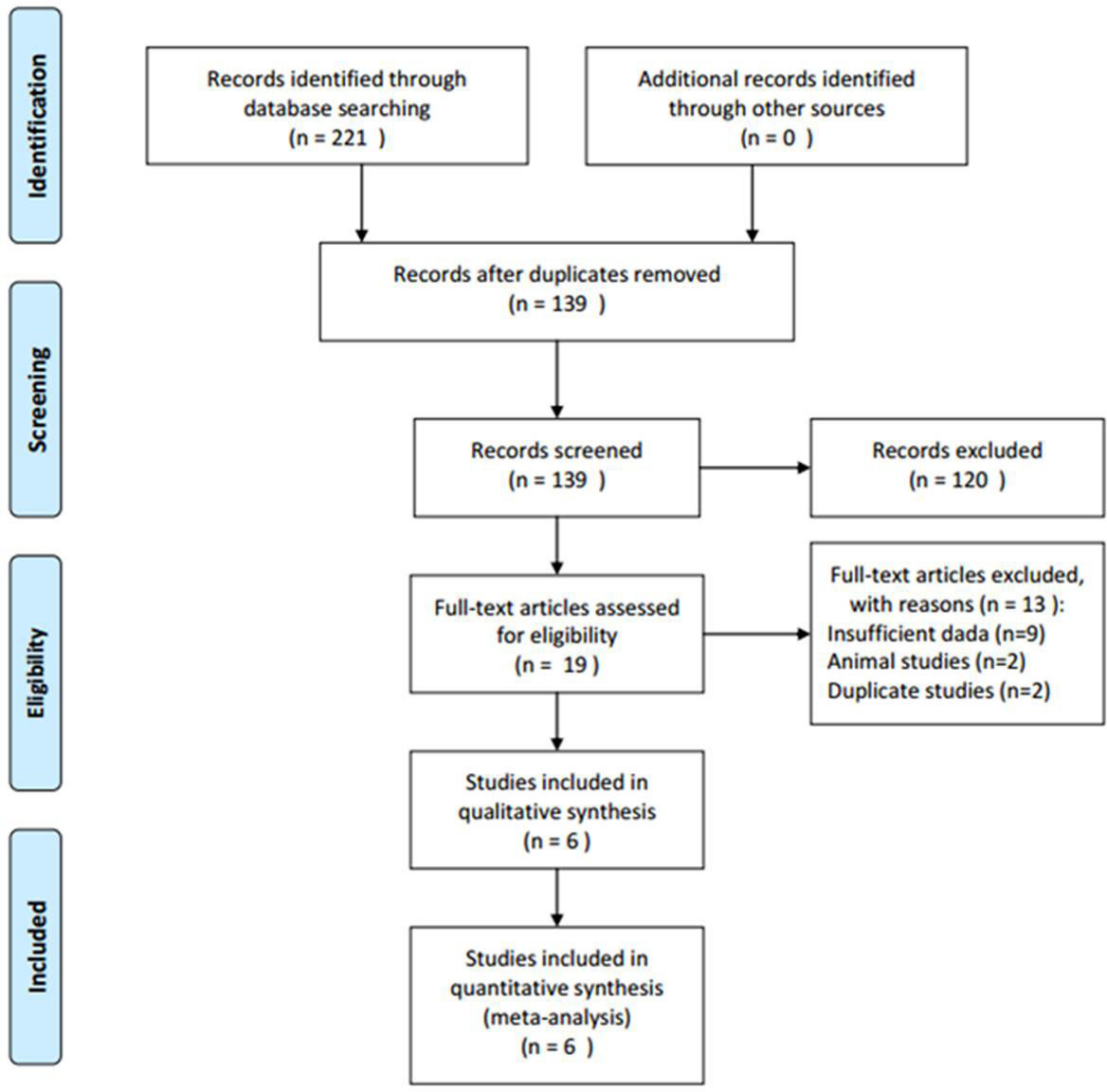
Flow chart for selection of studies

Initial literature search identified 221 items through aforementioned database and 139 records were screened by title and abstract after duplicate records were removed. Subsequently, 19 full-text articles were evaluated and 13 studies were excluded because they lacked necessary data, were animal studies or duplicate studies from the same patients. Finally, six studies [15–20] published from 2013 to 2016 were included for meta-analysis. The study selection process was shown in Figure 1. The total sample size was 575, ranging from 40 to 201, with a median value of 77.5. Two studies [15, 17] were conducted in China, one [16] was performed in Canada, one [18] was in Egypt, one [19] was in India and one [20] was in Germany, respectively. Four studies [15, 16, 18, 19] examined EZH2 expression by immunohistochemistry staining (IHC) and two [17, 20] used genetic testing. All six studies investigated the prognostic value of EZH2 for OS and three studies [16, 18, 19] explored the association between EZH2 and PFS. The detailed characteristics of included studies were depicted in Table 1.

**Table 1:** Characteristics of included studies into meta-analysis

Study	Year	Country	Patients (n)	Age(years) Mean(range)	Histological type	WHO grade	Therapy	Study duration	Survival outcomes	Method	Positive (%)	NOS score
Wu	2013	China	128	42(12–71)	Glioma	I-IV	Surgical resection	2000–2010	OS	IHC	62.5	8
Ailon	2015	Canada	201	7(0.4–15.8)	Glioma	II-III	Surgical resection	1986–2006	OS,PFS	IHC	NA	7
Zhang	2015	China	83	43	Glioblastoma	IV	Surgical resection	2006–2009	OS	Genetic testing	51.8	6
Ahmed	2016	Egypt	40	40(7–72)	Glioma	I-IV	Surgical resection	2011–2014	OS,PFS	IHC	55	7
Purkait	2016	India	51	NA	Glioblastoma	IV	Surgical resection	2009–2014	OS,PFS	IHC	94.3	7
Wiese	2016	Germany	72	3–21	Glioblastoma	IV	NA	NA	OS	Genetic testing	47.3	6

NA: not available; OS: overall survival; PFS: progression-free survival; IHC: immunohistochemistry staining; NOS score: the Newcastle-Ottawa Scale score.

### Prognostic value of EZH2 for OS and PFS

**Figure 2:**
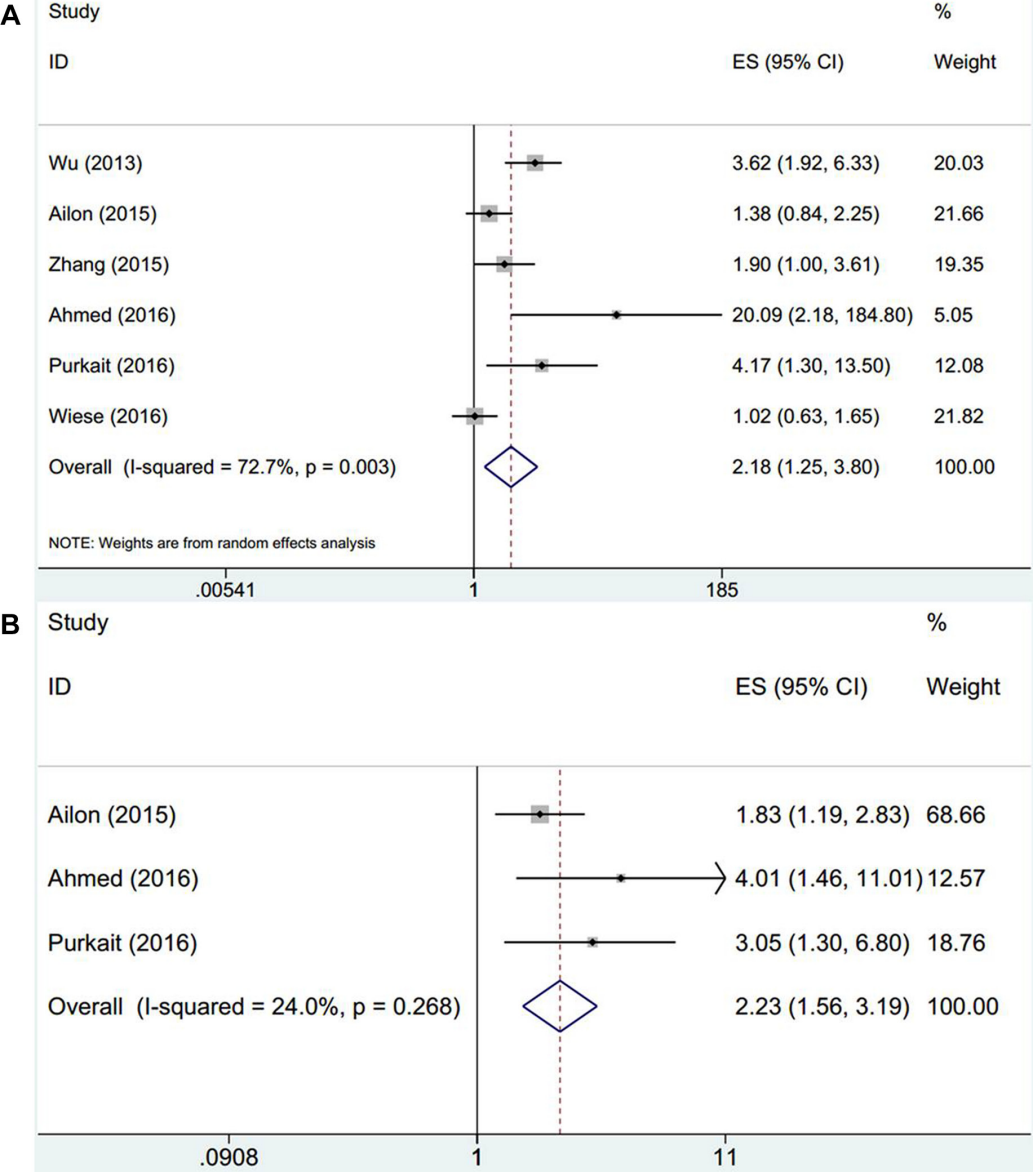
Forest plot of the association between EZH2 and (A) OS and (B) PFS in glioma patients

Pooled HR and 95% CI from 6 studies involving 575 patients was HR = 2.18, 95% CI: 1.25–3.8, *p* = 0.006 by random-effect model (Figure 2A, Table 2), indicating that EZH2 overexpression was correlated with poor OS. Moreover, combined HR and 95% CI for PFS was HR = 2.23, 95% CI: 1.56–3.19, *p* < 0.001, with moderate heterogeneity (Figure 2B, Table 2). Stratified analysis was conducted for further analysis, the results demonstrated that EZH2 still maintain prognostic value for poor OS in Asian patients (*n* = 3, HR = 2.83, 95% CI: 1.88–4.26, *p* < 0.001), for WHO grade I-IV (*n* = 3, HR = 3.07, 95% CI: 1.1–8.57, *p* = 0.033) and by IHC method (*n* = 4, HR = 3.19, 95% CI: 1.39–7.3, *p* = 0.006). However, EZH2 had no association with OS in non-Asian patients, WHO grade IV or by genetic testing (Table 2).

**Table 2:** Meta-analysis of EZH2 expression and survival of glioma patients

Factors	Studies (n)	Patients (n)	Effects model	HR (95%CI)	*p*-value	Heterogenicity	
						I^2^(%)	Ph
OS	6	575	Random	2.18 (1.25–3.8)	0.006	72.7	0.003
Region							
Asian countries	3	262	Fixed	2.83 (1.88–4.26)	< 0.001	21.9	0.278
Non-Asian countries	3	313	Random	1.56 (0.73–3.36)	0.253	70.9	0.032
WHO grade							
I-IV	3	369	Random	3.07 (1.1–8.57)	0.033	80	0.007
IV	3	206	Random	1.73 (0.86–3.39)	0.127	66.1	0.052
Method							
IHC	4	420	Random	3.19 (1.39–7.3)	0.006	72.9	0.011
Genetic testing	2	155	Random	1.34 (0.73–2.46)	0.344	57.2	0.126
PFS	3	292	Fixed	2.23 (1.56–3.19)	< 0.001	24	0.268

### Correlation between EZH2 and clinical characteristics in glioma

**Figure 3:**
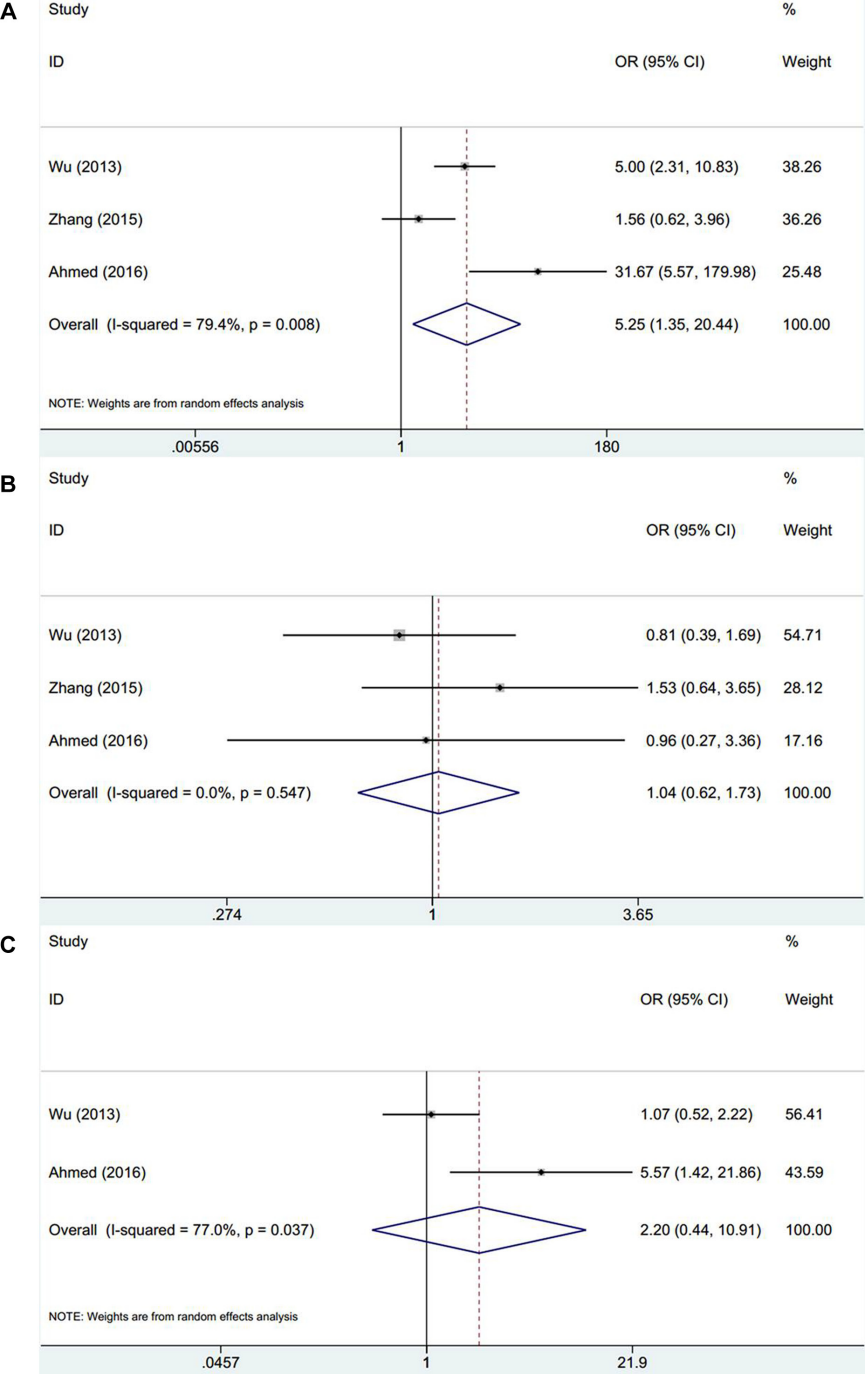
Meta-analysis of correlation between EZH2 expression and clinical factors in glioma (**A**) KPS score, (**B**) gender, and (**C**) age.

Three studies [15, 17, 18] exploited the relevance between EZH2 and gender as well as KPS score and two studies [15, 18] reported the association between EZH2 and age. The combined data showed that high EZH2 expression significantly associated with lower KPS score (*n* = 3, OR = 5.25, 95% CI: 1.35–20.44, *p* = 0.017, Table 3, Figure 3A) whereas EZH2 was not shown to be related with gender (*n* = 3, OR = 1.04, 95% CI: 0.62–1.73, *p* = 0.883, Table 3, Figure 3B) or age (*n* = 2, OR = 2.2, 95% CI: 0.44–10.91, *p* = 0.335, Table 3, Figure 3C).

**Table 3:** Association between EZH2 and clinicalpathological features in glioma patients

Factors	Studies (n)	Patients (n)	Effects model	OR (95%CI)	*p*-value	Heterogeneity	
						I^2^(%)	Ph
KPS score (< 80 vs ≥ 80)	3	251	Random	5.25(1.35–20.44)	0.017	79.4	0.008
Gender (male vs female)	3	251	Fixed	1.04(0.62–1.73)	0.883	0	0.547
Age (≥ 55 years vs < 55 years)	2	168	Random	2.2(0.44–10.91)	0.335	77	0.037

### Sensitivity analysis

**Figure 4:**
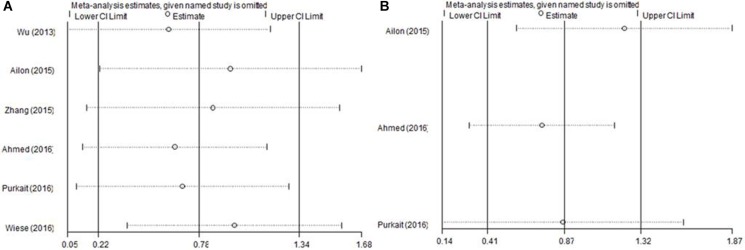
Sensitivity analysis of EZH2 and (A) OS and (B) PFS

Sensitivity analysis was conducted by omitting each study per time to check if individual study affected the final results. As shown in Figure 4, the overall results were not substantially altered, which confirmed the credibility of our results.

### Publication bias

**Figure 5:**
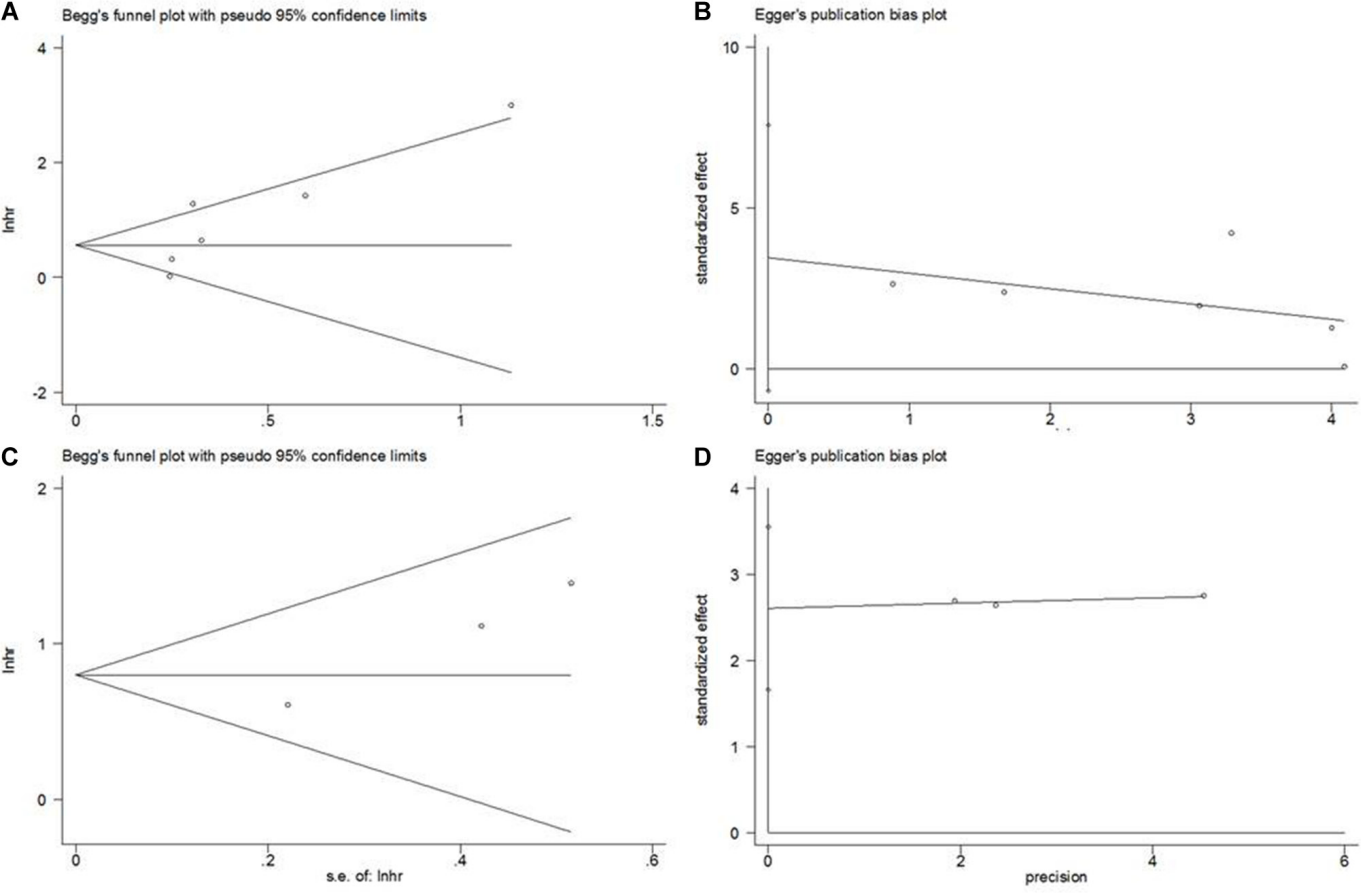
Publication bias test (**A**) Begg's funnel plot for OS, *p* = 0.133, (**B**) Egger's test for OS, *p* = 0.081; (**C**) Begg's funnel plot for PFS, *p* = 0.296; (**D**) Egger's test for PFS, *p* = 0.063.

Potential publication bias was tested using Begg's funnel plot and Egger's test. The results were summarized in Figure 5. The data demonstrated that there was no significant publication bias for all analyses, indicating no evidence of significant publication bias.

## DISCUSSION

The biological role and prognostic significance of EZH2 were extensively investigated in gliomas [21–26]. Regarding the prognostic value, various studies presented controversial results, which may confuse clinicians for selecting EZH2 for prognostication. In the current meta-analysis, we extracted data from six eligible studies comprising 575 patients and pooled data for analysis. The combined results showed that EZH2 predicted poor OS and PFS in glioma patients. In addition, elevated EZH2 expression still had significant prognostic role for shorter OS in Asian patients, for WHO grade I-IV glioma and using IHC detection method. As for clinical relevance, EZH2 was found to be related with KPS score < 80, while had no correlation to age or gender. Furthermore, sensitivity analysis and publication bias examination illustrated the robustness of our study. To our knowledge, the present study was the first meta-analysis to explore the prognostic value of EZH2 in glioma patients.

EZH2 is a multifaceted oncogenic protein which facilitates tumor occurrence and proliferation mainly through methylation of tumor suppressor genes [7]. Recent studies demonstrated that EZH2 could contribute to glioma progression by more extensive mechanisms. Pang *et al.* [27] showed that EZH2 could activate EAF2-HIF1α signaling pathway to confer Warburg effect, which provides cancer cells glycolysis for energy metabolism. In addition, Zhang *et al.* [28] showed that EZH2, coordinating with long non-coding RNA (lncRNA) Hox transcript antisense intergenic RNA (HOTAIR), could accelerate cell cycle progression in glioma. What's more, EZH2 could upregulate STAT3 activity by methylating STAT3, leading to glioblastoma stem-like cells (GSCs) formation, further to sustain the stemness of GSCs [29]. This evidence, along with the findings of our meta-analysis, may help to reveal the multiple roles of EZH2 in glioma development and to establish EZH2 as a useful biomarker for prognosis.

We have found that a variety of studies investigated the prognostic significance of EZH2 in diverse cancer types by meta-analysis [30–34]. Guo *et al.* [30] found that EZH2 had positive association with TNM stage and lymph node metastasis in gastric cancer. Wang and colleagues showed that EZH2 was predictive for poor OS in breast cancer patients [33]. In the current meta-analysis, we revealed that EZH2 was associated with KPS score < 80, which indicated the suboptimal condition of patients. Our results were in line with previous studies. Notably, we found that the included studies recruited patients with different glioma entities. Because gliomas include diverse subtypes, and some subtypes occur mainly in children. Therefore, the included studies with different age groups may introduce selection bias, which call for further investigations on separate patient groups.

Despite this was the first meta-analysis regarding EZH2 in glioma, there were several limitations should be pointed out. First, the sample size in this study was relatively small, which hinders further analyses of EZH2 and clinical features because limited data was provided. Second, heterogeneity is a potential problem existed in this study. Although we adopted random-effects model when significant heterogeneity was detected, the inherent heterogeneity of included studies still existed. Third, only 6 studies were included in this meta-analysis, although Begg's test did not detect significant publication bias, it is due to Begg's test was suboptimal to detect publication when the included studies are less than 25 [35]. Therefore, potential publication bias may still exist in this meta-analysis. These limitations may undermine the reliability of this study; however as these limitations were mostly inherent characteristics of meta-analysis studies. From a statistical point of view, we at least provide evidence that EZH2 is a potential prognostic marker for glioma. Notably, the results should be further validated in clinical settings.

In summary, through performing a meta-analysis, we identified EZH2 overexpression as a prognostic marker for poor OS and PFS in patients with glioma, especially in Asian patients, for WHO grade I-IV and when using IHC method. EZH2 was also found to be related to KPS score < 80. Our results provide the evidence that EZH2 might be applied as a potential biomarker for glioma. Due to several limitations, further studies are required to verify the conclusion of the present meta-analysis.

## MATERIALS AND METHODS

### Literature search and selection criteria

The current meta-analysis was carried out according to Preferred Reporting Items for Systematic Reviews and Meta-Analyses (PRISMA) statement [36]. Electronic databases Pubmed, Embase and Web of Science were thoroughly searched and the last search was on July, 2016. The following keywords and Medical Subject Headings (MeSH) trems were used: “glioma” (MeSH term), “gliomas”, “EZH2” and “Enhancer of zeste homologue 2”. Reference lists were also reviewed for additional studies.

Eligible studies need to meet the following inclusion criteria:(1) the diagnosis of glioma was pathologically confirmed; (2) EZH2 expression was evaluated in glioma tissues; (3) EZH2 expression was detected by any method; (4) the association between EZH2 and clinical features or survival outcomes in glioma patients were reported; (5) hazard ratio (HR) and 95% confidence interval (CI) were directly reported in text or sufficient data was provided for HR and 95%CI calculation by Tierney's method [37]; (6) full-text articles published in English. Studies were discarded if they met any one of the exclusion criteria:(1) conference abstracts, reviews, letters and case reports; (2)nonhuman studies; (3) published not in English; (4) if different articles were published based on the same patient group, the most comprehensive one was selected.

### Data extraction and study quantity assessment

Two investigators independently extracted the following information using a standard form: first author's name, publication year, country, number of patients, WHO grade, EZH2 detection method, study period, study design and survival outcomes. Disagreements between the two investigators were settled by discussion. The quality of included studies was also assessed by using Newcastle-Ottawa Scale (NOS) scale [38]. On basis of three categories: selection, comparability and outcome, a single was evaluated with a full score of 9 and studies assigned > 6 was considered as high quality studies.

### Statistical analysis

To assess the association between EZH2 expression and survival outcomes including OS and progression-free survival (PFS), HRs and 95% CIs were utilized to combine as the effective value. Heterogeneity among studies was calculated using inconsistency index (Ι^2^) statistic and Q statistic. I^2^ > 50% or p for heterogeneity (P_h_) < 0.1 indicate significant heterogeneity and a random-effect model was used, otherwise, a fixed-effect model was used. Subgroup analysis was conducted for further investigation. Combined odd ratios (ORs) and 95% CIs were calculated to examine the relationship between EZH2 and clinical features. Sensitivity analysis by serial omission of each study was performed to test the influence of single study on the overall results. Publication bias was qualitatively evaluated by applying Begg's test and Egger's test. All statistical analysis was performed using Stata software version 12.0 (Stata, College Station, TX). *P* < 0.05 was considered as statistically significant.

## References

[1] Ferlay J, Shin HR, Bray F, Forman D, Mathers C, Parkin DM (2010). Estimates of worldwide burden of cancer in 2008: GLOBOCAN 2008. Int J Cancer.

[2] Schwartzbaum JA, Fisher JL, Aldape KD, Wrensch M (2006). Epidemiology and molecular pathology of glioma. Nat Clin Pract Neurol.

[3] Woolf EC, Scheck AC (2012). Metabolism and glioma therapy. CNS Oncol.

[4] Davis FG, McCarthy BJ, Berger MS (1999). Centralized databases available for describing primary brain tumor incidence, survival, and treatment: Central Brain Tumor Registry of the United States; Surveillance, Epidemiology, and End Results; and National Cancer Data Base. Neuro Oncol.

[5] Chinaranagari S, Sharma P, Chaudhary J (2014). EZH2 dependent H3K27me3 is involved in epigenetic silencing of ID4 in prostate cancer. Oncotarget.

[6] Wang CX, Liu ZH, Woo CW, Li ZJ, Wang LF, Wei JS, Marquez VE, Bates SE, Jin QH, Khan J, Ge K, Thiele CJ (2012). EZH2 Mediates Epigenetic Silencing of Neuroblastoma Suppressor Genes CASZ1, CLU, RUNX3, and NGFR. Cancer research.

[7] Chang CJ, Hung MC (2012). The role of EZH2 in tumour progression. Br J Cancer.

[8] Lu C, Han HD, Mangala LS, Ali-Fehmi R, Newton CS, Ozbun L, Armaiz-Pena GN, Hu W, Stone RL, Munkarah A, Ravoori MK, Shahzad MM, Lee JW (2010). Regulation of tumor angiogenesis by EZH2. Cancer Cell.

[9] Huqun Ishikawa R, Zhang JL, Miyazawa H, Goto Y, Shimizu Y, Hagiwara K, Koyama N (2012). Enhancer of zeste homolog 2 is a novel prognostic biomarker in nonsmall cell lung cancer. Cancer.

[10] Xu KX, Wu ZJ, Groner AC, He H, Cai CM, Lis RT, Wu XQ, Stack EC, Loda M, Liu T, Xu H, Cato L, Thornton JE (2012). EZH2 Oncogenic Activity in Castration-Resistant Prostate Cancer Cells Is Polycomb-Independent. Science.

[11] Cao W, Feng ZE, Cui ZB, Zhang CP, Sun ZY, Mao L, Chen WT (2012). Up-regulation of enhancer of zeste homolog 2 is associated positively with cyclin D1 overexpression and poor clinical outcome in head and neck squamous cell carcinoma. Cancer.

[12] Chang CJ, Yang JY, Xia WY, Chen CT, Xie XM, Chao CH, Woodward WA, Hsu JM, Hortobagyi GN, Hung MC (2011). EZH2 Promotes Expansion of Breast Tumor Initiating Cells through Activation of RAF1-beta-Catenin Signaling. Cancer Cell.

[13] Zhuang C, Wang P, Huang D, Xu L, Wang X, Wang L, Hu L (2016). A double-negative feedback loop between EZH2 and miR-26a regulates tumor cell growth in hepatocellular carcinoma. Int J Oncol.

[14] He LJ, Cai MY, Xu GL, Li JJ, Weng ZJ, Xu DZ, Luo GY, Zhu SL, Xie D (2012). Prognostic significance of overexpression of EZH2 and H3k27me3 proteins in gastric cancer. Asian Pac J Cancer Prev.

[15] Wu Z, Wang Q, Wang L, Li G, Liu H, Fan F, Li Z, Li Y, Tu Y (2013). Combined aberrant expression of Bmi1 and EZH2 is predictive of poor prognosis in glioma patients. J Neurol Sci.

[16] Ailon T, Dunham C, Carret AS, Tabori U, McNeely PD, Zelcer S, Wilson B, Lafay-Cousin L, Johnston D, Eisenstat DD, Silva M, Jabado N, Goddard KJ (2015). The role of resection alone in select children with intracranial ependymoma: the Canadian Pediatric Brain Tumour Consortium experience. Childs Nerv Syst.

[17] Zhang J, Chen L, Han L, Shi Z, Zhang J, Pu P, Kang C (2015). EZH2 is a negative prognostic factor and exhibits pro-oncogenic activity in glioblastoma. Cancer Lett.

[18] Ahmed S, Rashed H, Hegazy A, Mohamed AM, Elmesallamy W (2016). Prognostic Value of ALDH1, EZH2 and Ki-67 in Astrocytic Gliomas. Turk Patoloji Derg.

[19] Purkait S, Sharma V, Kumar A, Pathak P, Mallick S, Jha P, Sharma MC, Suri V, Julka PK, Suri A, Sharma BS, Sarkar C (2016). Expression of DNA methyltransferases 1 and 3B correlates with EZH2 and this 3-marker epigenetic signature predicts outcome in glioblastomas. Exp Mol Pathol.

[20] Wiese M, Schill F, Sturm D, Pfister S, Hulleman E, Johnsen SA, Kramm CM (2016). No Significant Cytotoxic Effect of the EZH2 Inhibitor Tazemetostat (EPZ-6438) on Pediatric Glioma Cells with Wildtype Histone 3 or Mutated Histone 3. 3. Klin Padiatr.

[21] Orzan F, Pellegatta S, Poliani PL, Pisati F, Caldera V, Menghi F, Kapetis D, Marras C, Schiffer D, Finocchiaro G (2011). Enhancer of Zeste 2 (EZH2) is up-regulated in malignant gliomas and in glioma stem-like cells. Neuropathol Appl Neurobiol.

[22] Ahani N, Shirkoohi R, Rokouei M, Alipour Eskandani M, Nikravesh A (2014). Overexpression of enhancer of zeste human homolog 2 (EZH2) gene in human cytomegalovirus positive glioblastoma multiforme tissues. Med Oncol.

[23] Kim SH, Joshi K, Ezhilarasan R, Myers TR, Siu J, Gu C, Nakano-Okuno M, Taylor D, Minata M, Sulman EP, Lee J, Bhat KP, Salcini AE (2015). EZH2 protects glioma stem cells from radiation-induced cell death in a MELK/FOXM1-dependent manner. Stem Cell Reports.

[24] Li AM, Dunham C, Tabori U, Carret AS, McNeely PD, Johnston D, Lafay-Cousin L, Wilson B, Eisenstat DD, Jabado N, Zelcer S, Silva M, Scheinemann K (2015). EZH2 expression is a prognostic factor in childhood intracranial ependymoma: a Canadian Pediatric Brain Tumor Consortium study. Cancer.

[25] Lin L, Zheng Y, Tu Y, Wang Z, Liu H, Lu X, Xu L, Yuan J (2015). MicroRNA-144 suppresses tumorigenesis and tumor progression of astrocytoma by targeting EZH2. Hum Pathol.

[26] Sharma V, Purkait S, Takkar S, Malgulwar PB, Kumar A, Pathak P, Suri V, Sharma MC, Suri A, Kale SS, Kulshreshtha R, Sarkar C (2016). Analysis of EZH2: micro-RNA network in low and high grade astrocytic tumors. Brain Tumor Pathol.

[27] Pang B, Zheng XR, Tian JX, Gao TH, Gu GY, Zhang R, Fu YB, Pang Q, Li XG, Liu Q (2016). EZH2 promotes metabolic reprogramming in glioblastomas through epigenetic repression of EAF2-HIF1alpha signaling. Oncotarget.

[28] Zhang K, Sun X, Zhou X, Han L, Chen L, Shi Z, Zhang A, Ye M, Wang Q, Liu C, Wei J, Ren Y, Yang J (2015). Long non-coding RNA HOTAIR promotes glioblastoma cell cycle progression in an EZH2 dependent manner. Oncotarget.

[29] Kim E, Kim M, Woo DH, Shin Y, Shin J, Chang N, Oh YT, Kim H, Rheey J, Nakano I, Lee C, Joo KM, Rich JN (2013). Phosphorylation of EZH2 activates STAT3 signaling via STAT3 methylation and promotes tumorigenicity of glioblastoma stem-like cells. Cancer Cell.

[30] Guo L, Yang TF, Liang SC, Guo JX, Wang Q (2014). Role of EZH2 protein expression in gastric carcinogenesis among Asians: a meta-analysis. Tumour Biol.

[31] Chen S, Huang L, Sun K, Wu D, Li M, Li M, Zhong B, Chen M, Zhang S (2015). Enhancer of zeste homolog 2 as an independent prognostic marker for cancer: a meta-analysis. PLoS One.

[32] Wang W, Wang F, Zong G, Liu R, Zhang Y, Luan Y, Xu L, Xuan J (2015). Prognostic significance of EZH2 expression in patients with digestive cancers: a meta-analysis. Int J Clin Exp Med.

[33] Wang X, Hu B, Shen H, Zhou H, Xue X, Chen Y, Chen S, Han Y, Yuan B, Zhao H, Zhi Q, Kuang Y (2015). Clinical and prognostic relevance of EZH2 in breast cancer: A meta-analysis. Biomed Pharmacother.

[34] Jiang T, Wang Y, Zhou F, Gao G, Ren S, Zhou C (2016). Prognostic value of high EZH2 expression in patients with different types of cancer: a systematic review with meta-analysis. Oncotarget.

[35] Begg CB, Mazumdar M (1994). Operating characteristics of a rank correlation test for publication bias. Biometrics.

[36] Moher D, Liberati A, Tetzlaff J, Altman DG (2009). Preferred reporting items for systematic reviews and meta-analyses: the PRISMA statement. PLoS Med.

[37] Tierney JF, Stewart LA, Ghersi D, Burdett S, Sydes MR (2007). Practical methods for incorporating summary time-to-event data into meta-analysis. Trials.

[38] Stang A (2010). Critical evaluation of the Newcastle-Ottawa scale for the assessment of the quality of nonrandomized studies in meta-analyses. Eur J Epidemiol.

